# Identifying the potential genes in alpha synuclein driving ferroptosis of Parkinson’s disease

**DOI:** 10.1038/s41598-023-44124-4

**Published:** 2023-10-06

**Authors:** Min Wang, Taole Li, Rong Gao, Yu Zhang, Yanqing Han

**Affiliations:** 1 Department of Neurology, Shanxi Cardiovascular Hospital/Cardiovascular Hospital Affiliated to Shanxi Medical University, Taiyuan, Shanxi China; 2 Department of Neurology, Xiangya Hospital, Central South University, Changsha, Hunan China; 3 Hunan Key Laboratory of Pharmacogenetics, Department of Clinical Pharmacology, Institute of Clinical Pharmacology, Xiangya Hospital, Central South University, Changsha, Hunan China

**Keywords:** Neurodegeneration, Neurodegenerative diseases

## Abstract

Parkinson’s disease (PD) is a common neurodegenerative disease with aggregation of α-synuclein (α-syn) in substantia nigra (SN). The association between the α-syn and ferroptosis in PD remains unclear. GSE49036 was obtained from the Gene Expression Omnibus (GEO) database and intersected with ferroptosis genes. Bioinformatics analysis was used to identify the potential differentially expressed genes (DEGs) included the development of Gene set enrichment analysis (GSEA), Kyoto Encyclopedia of Genes and Genomes (KEGG) and protein–protein interaction (PPI) network. We screened 8 key genes were modulated and crosslinked by 238 miRNAs. Additionally, 5 hub genes were predicted and 38 lncRNAs targeting 3 key miRNAs were revealed. Finally, 3 hub genes (PIK3CA, BRD4, ATM) and the key lncRNA (NEAT1) were verified in neurotoxic PD models. The in vitro experiments showed that PIK3CA and ATM were significantly upregulated or the BRD4 was downregulated in the rotenone treatment and they could be rescued by the specific ferroptosis inhibitor, liproxstatin-1. The expression of the key lncRNA NEAT1 were consistent with the hub genes in same models. This study identified the proposed NEAT1-PIK3CA/ATM ceRNA network may be a specific biomarker in α-syn driving ferroptosis as well as to predict clinical outcomes and therapeutic targets in PD patients.

## Introduction

Parkinson’s disease (PD) is the second most common neurodegenerative disease characterized by motor or non-motor symptoms such as bradykinesia, tremor, constipation, psychiatric disorders and sleep disturbances^1^. The main pathological features of PD are the progressive death of dopaminergic neurons in substantia nigra pars compacta (SNpc) and the intraneuronal Lewy bodies formed with aggregated α-synuclein. *SNCA* is one of the PD-associated genes encodes the human α-synuclein protein, missense mutation in it may cause the familial or sporadic forms of the disease^2^. Accumulating evidence suggest that the α-synuclein, as the soluble oligomers that intricately impairing many aspects of subcellular functions including oxidative stress, neuroinflammation, mitochondrial or lysosomal homeostasis imbalance. It also implicated in multiple cell death mechanisms such as apoptosis, autophagy, necroptosis, pyroptosis and ferroptosis^3^.

Ferroptosis is a distinct identified form of cell death with key features of the highly iron-dependent, lipid peroxidation and GPX4 depletion^4,5^. Subsequent studies revealed ferroptosis has been clear potential linked to PD, brain iron overloaded, PUFAs composition altered or GSH and xCT decreased are hallmarkers in PD pathogenesis^6,7^. In addition, α-synuclein as a key contributor to ferroptosis progression that is considered as not only regulate the iron transport but also modulate the lipid metabolism in dopaminergic neurons^8^. However, the exact mechanisms and physiological function of α-synuclein linking to the ferroptosis remains unclear, further genome wide association studies are needed.

There have been no bioinformatics-based studies on the mechanism of ferroptosis related genes with α-synuclein in PD. In the present study, we first used data sets from the Gene Expression Omnibus (GEO) database to screen differentially expressed genes (DEGs) in PD patients. These DEGs were intersected with the ferroptosis dataset (FerrDb) to obtain the hub genes. We also performed enrichment analysis to underlying mechanisms. Moreover, we investigated the key miRNAs and lncRNAs that may contribute to PD progress and constructed a competing endogenous RNA (ceRNA) network. Finally, the above-mentioned hypothesis was validated by the experiment test with PD models. Our results will provide a new angle to uncover the ferroptosis after α-synuclein aggregated in PD patients and new thoughts for diagnosis and treatment of α-synucleinopathy.

## Materials and methods

### Data acquisition and processing

We obtained the PD patients mRNA expression profile data (GSE49036) from NCBI GEO database^9^. The microarray dataset consisted of 16 post-mortem substantia nigra (SN) samples of PD donors, involving 8 control samples with Braak α-synuclein Stage 0 and 8 PD samples with Braak α-synuclein Stage 5–6. The dataset was used for further analysis and mining.

### DEGs analysis

GEO2R, an online tool, was used to analyse the differential expression between the control samples and PD samples to identify the DEGs (the criteria was set to log2|FC|> 0.2 and adjusted *p* value < 0.05). We also downloaded the dataset that included 259 genes from the FerrDb database and intersected it with GSE49036 to identify the DEGs. To directly show the harvested DEGs mentioned above, volcano map, heatmap and Venn diagram were drawn by using the online tools GEO2R, HiPlot and Venny2.1.0 respectively.

### GSEA analysis

Gene set enrichment analysis (GSEA) was used to sort genes according to the degree of DEGs in GSE49036. The GSEA of WebGestalt, an online software that filtered the gene sets according to the number of genes contained, with a minimum number of 7 genes and a maximum number of 2000 genes per gene set by default, was operated to investigate involved Kyoto Encyclopedia of Genes and Genomes (KEGG) pathways^10–12^ of the DEGs. Adjusted *p* < 0.05 and FDR ≤ 0.05 was considered as significant enrichment.

### Functional enrichment analysis

Gene Ontology (GO) function and KEGG pathway enrichment analysis of DEGs in PD were operated using the HiPlot and Metascape, two online enrichment analysis tools with different algorithms. The reference gene sets were performed with GO-BP (biological process), GO-CC (cell components) and GO-MF (molecular function). The results with adjusted *p* < 0.05 and FDR ≤ 0.05 were regarded as the thresholds for selecting significant GO terms and for KEGG pathway analysis.

### PPI network construction

The DEGs were processed using the STRING database, an online tool that retrieved the interaction between proteins. We used to predict the correlation among the DEGs (confidence score > 0.4) and the interaction relationship was visualized by Cytoscape v3.9.1 software. The nodes represented proteins and the edges represented the links between them. Moreover, the molecular complex detection (MCODE) and the CytoHubba, two plugins of Cytoscape were carried out to screen hub genes. The MCODE was used for clustering analysis of key gene sub-networks and the CytoHubba was employed to select the top 10 hub genes via 11 maximal clique centrality (MCC) methods for the further study.

### Gene-miRNA network construction

The hub 9 genes, selected according to the PPI network, were analysed to predict targeted pivotal miRNAs and build the gene-miRNA interaction relationship networks. We applied miRWalk 2.0 to integrate miRNA databases and intersected the predicted results of the miRDB database to ensure the accuracy of the results. We visualized the networks by employing Cytoscape v3.9.1 and analysed their biological pathways by Funrich, an functional enrichment analysis tool special for miRNAs.

### miRNA–lncRNA prediction and ceRNA network construction

Based on StarBase v2.0, the upstream target lncRNAs of the predicted miRNAs were identified and then intersected with the differential lncRNAs. We selected the cross-linked hub genes showing the interaction of the miRNAs relevant lncRNAs. The highest reliability (very high stringency, > 5) was selected as the criterion. The ceRNA network of the hub genes-miRNAs–lncRNAs was constructed by the Cytoscape v3.9.1 software.

### Cell cultures and drug treatment

Human neuroblastoma SH-SY5Y cells (#CL-0208) were purchased from Procell (Wuhan, China) and cultured in Minimum Eagle Medium (MEM, Procell, Wuhan, China) supplemented with 10% fetal bovine serum, 1% penicillin and streptomycin. Cells were incubated at 37 °C and 95% humidity with 5% CO_2_. To contribute PD cell culture model in vitro, SH-SY5Y cells were seeded in 1 ml media at a density of 1 × 10^5^ per well in a 12-well plate for approximately 24 h., then were treated with indicated drugs respectively. In control group, cells were exposed to medium (containing 0.1% dimethyl sulfoxide). In model group, cells were treated with 1 µmol/L (µM) rotenone (#S2348, Selleck, Houston, USA) with or without 5 µmol/L (µM) liproxstatin-1 (#S7699, Selleck, Houston, USA). After being treated with 24 h, the cells were digested and washed with phosphate buffer saline and subjected to further treatment and analysis.

### RNA extraction and quantitative real time-PCR (qRT-PCR) analysis

The total SH-SY5Y cell RNA was extracted using the TRIzol reagent, the quality was tested using Quantus Fluorometer/spectrometer and the assessment of purity relied on the A260/A280 ratio. The cDNA synthesis was performed using reverse transcription reaction kit (Vazyme) following the manufacturer’s instructions. Quantitative real-time PCR was carried out using the Hieff Universal qPCR SYBR Green Master Mix (UNICON, Yeasen Biotechnology, Shanghai, China). Primer sequences used for qRT-PCR assays were listed in Table 1. Relative expression levels of hub genes or lncRNAs were calculated by 2−△△CT method normalized to GAPDH compared with control samples.

**Table 1:** Specific primers used for quantitative qRT-PCR.

Gene	Forward (5′-3′)	Reverse (5′-3′)
PIK3CA	CCACGACCATCATCAGGTGAA	CCTCACGGAGGCATTCTAAAGT
ATM	ATCTGCTGCCGTCAACTAGAA	GATCTCGAATCAGGCGCTTAAA
BRD4	GAGCTACCCACAGAAGAAACC	GAGTCGATGCTTGAGTTGTGTT
NEAT1_1	GCGAGGTGCCTTTACTACAT	TGGAACCCAGAAGACAGA
NEAT1_2	TCCGAGGAAGATGTAAGG	TCTGTGGAATGAGGCAAC
GAPDH	ACAGCCTCAAGATCATCAGC	GGTCATGAGTCCTTCCACGAT

### Western blot analysis

Protein was extracted from SH-SY5Y cells using lysis buffer. For western blot analysis, equal amounts of protein were separated using 12% SDS–PAGE gels and transferred onto PVDF membranes by electroblotting. After blocking with 5% fat-free milk for 1 h, the membranes were incubated with primary antibodies α-synuclein (#SAB4502829, SigmaAldrich, Louis, MO, USA), GPX4 (#ab125066, Abcam, Cambridge, USA), FTH1 (#4393, Cell Signaling Technology, Danvers, USA) overnight at 4 °C and followed by appropriate species of HRP-conjugated secondary antibodies at 37 °C for 1 h. Finally, proteins were visualized by ECL reagents detection and the immunoreactive bands were quantified using the Image J software.

### Lipid peroxidation, GSH and iron content assay

SH-SY5Y cells were seeded in 12 well plates at a density of 1 × 10^5^ cells/well and treated with indicated drugs: normal group and rotenone group with or without liproxstatin-1. The cells from each sample were washed by PBS buffer, then homogenized and harvested by centrifugation at 1000 rpm at 4 °C for 5 min. The quantifications of lipid peroxidation (MDA) (#A003-1, Jiancheng Bioengineering Insitute, Nanjing, China), GSH (#A006-1-1, Jiancheng Bioengineering Insitute, Nanjing, China) and iron (#ab83366, Abcam, Cambridge, USA)in the collected supernatant were performed respectively with commercial assay kits according to the manufacturer’s instructions.

### Statistical analysis

All data are presented as mean ± standard deviation (SD). Two-tailed Student’s t test or one-way ANOVA followed by Tukey’s post hoc analysis was used to compare the differences between groups. Each experiment was replicated at least three times. Statistical analysis and visualization were performed using GraphPad Prism 8. The significance of differences was set at the level of *p* < 0.05.

### Ethics statement

All the information of patients were recruited from a public database and the study involving none of ethics issue.

## Results

### Identification of DEGs

**Figure 1:**
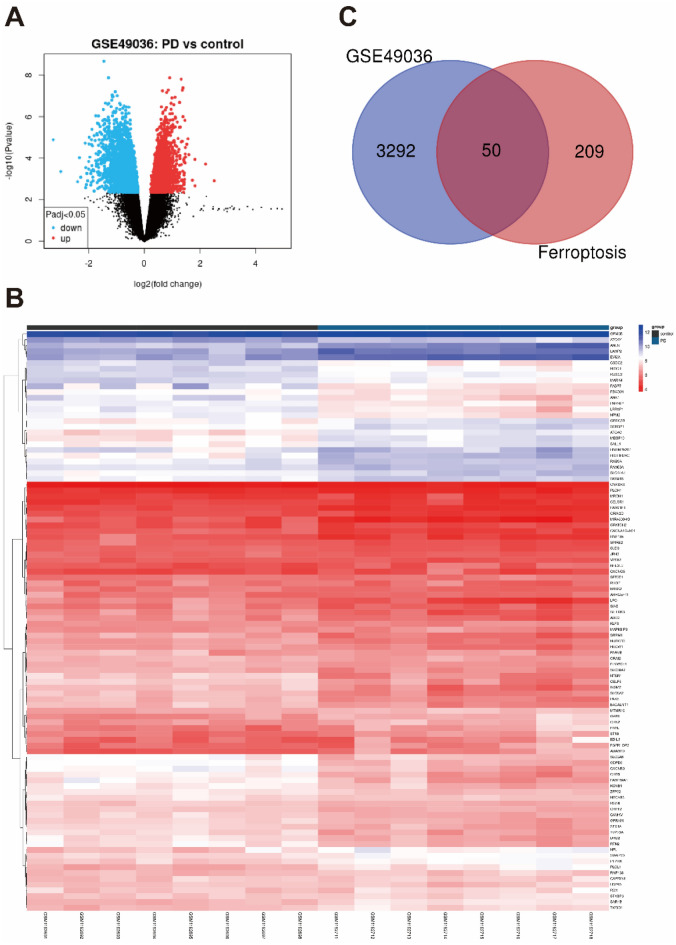
Identification of DEGs. (**A**)Volcano plot of DEGs between PD patients and controls (PD vs Control). (**B**) A heatmap of top 100 DEGs. (**C**) Venn diagram of ferroptosis DEGs by the GSE49036 intersected with FerrDB.

In total, the mRNA expression data of 16 substantia nigra tissue generated from GSE49036 with 8 PD patients (Braak α-synuclein Stage 5–6) and 8 controls (Braak α-synuclein Stage 0) were obtained. Compared with normal samples, a total of 3342 DEGs in the dataset were found (Fig. 1A,B). We also gained the dataset including 259 genes from FerrDb and intersected them with GSE49036 to identify 50 ferroptosis DEGs (Fig. 1C). Among the DEGs, 27 up-regulated and 23 down-regulated DEGs in the substantia nigra tissue were identified and they were further classified as ferroptosis driver, ferroptosis suppressor, ferroptosis marker or unclassified via the FerrDb online tool (Tables 2, 3).

**Table 2:** Ferroptosis DEGs of PD.

Up-regulate (27)	Down-regulate (23)
LAMP2, NFE2L2, ENPP2, ATG3	SLC2A6, CHAC1, ZFP69B, ACVR1B
PSAT1, ATM, FTL, ELAVL1	NF2, STMN1, ABCC1, ULK1
HMOX1, RB1, STAT3, BACH1	TFR2, HRAS, SLC2A8, MTOR
HMGB1, MTDH, KLHL24, ARNTL	BAP1, PML, GLS2, PHKG2
LPIN1, GCLC, PIK3CA, HSD17B11	NRAS, G6PD, ATG4D, MAFG
SCP2, AKR1C1, ATF3, FTH1	BRD4, MAP1LC3A, MAPK9
AKR1C3, BID, SLC7A11

**Table 3:** The ferroptosis DEGs were classified as driver, suppressor, marker and unclassified (*indicate the genes are also ferroptosis marker).

Driver (25)	Suppressor (15)	Marker (9)	Unclassified (1)
BID, ULK1, BACH1	AKR1C3, MTOR	PSAT1	HMOX1
ACVR1B, SCP2, HRAS	BRD4, FTH1*	SLC2A6	
LPIN1, MAP1LC3A, G6PD	ENPP2,	SLC2A8	
TFR2, MTDH, PHKG2	SLC7A11*	MAFG	
ELAVL1*, ATG3, ATF3*	PML, AKR1C1	HSD17B11	
NRAS, GLS2, MAPK9	LAMP2, NFE2L2*	ZFP69B	
BAP1, CHAC1*, ABCC1	ARNTL, STAT3	STMN1	
ATG4D, PIK3CA, HMGB1*,	GCLC, RB1, NF2	KLHL24	
ATM		FTL	

### GSEA analysis of DEGs

**Figure 2:**
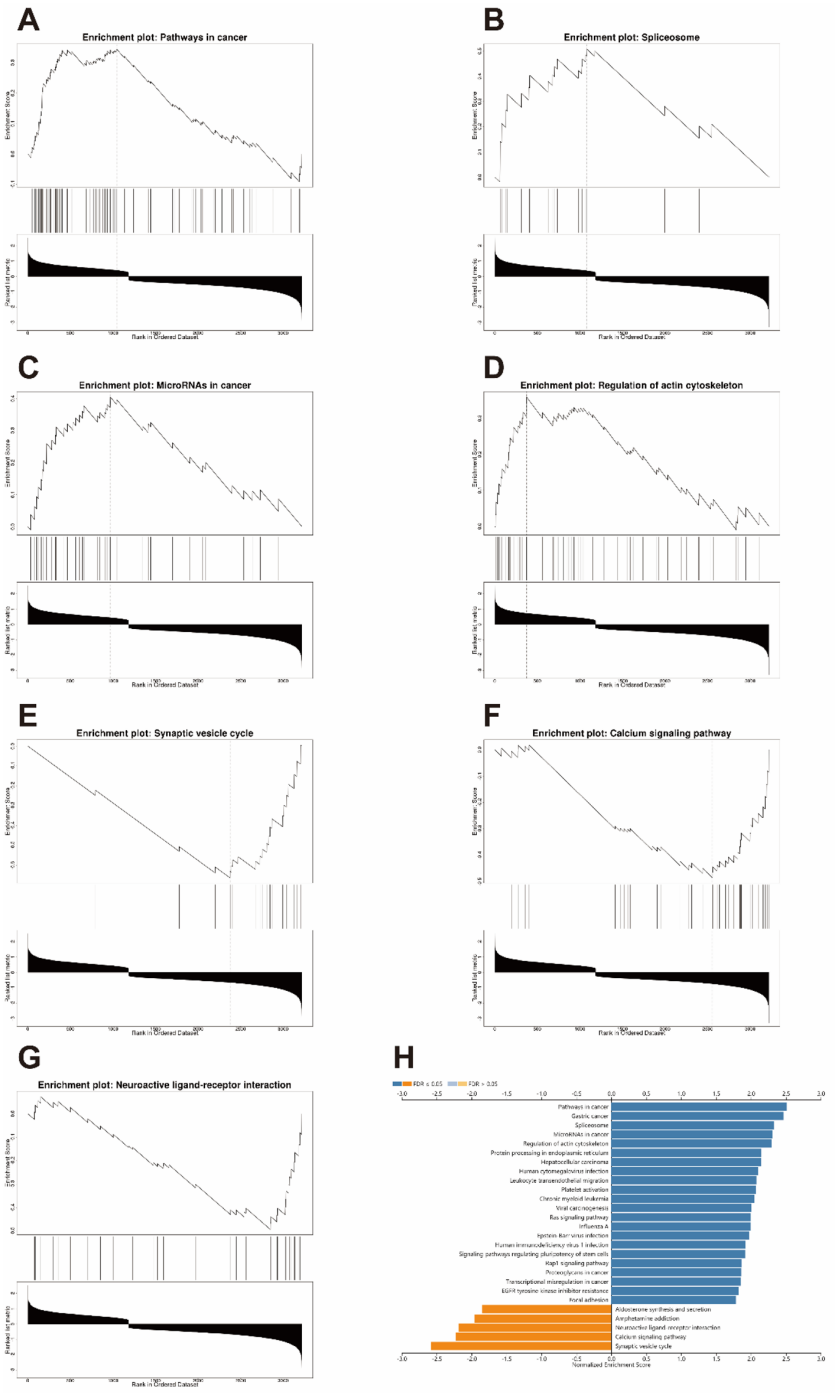
GSEA analysis of DEGs. (**A**–**G**) The result of the enrichment gene dataset analysis with the WebGestalt software. (**H**) The bar chart of the top biological pathways.

We used GSEA enrichment analysis in the KEGG pathway by WebGestalt software to uncover the underlying mechanisms of the DEGs. The results indicated that DEGs were mainly enriched in the various cancer pathways included microRNAs in cancer pathway. In addition, they were also significantly enriched in splicesome, regulation of actin cytoskeleton, synaptic vesicle cycle, calcium signaling pathway and neuroactive ligand-receptor interaction (Fig. 2).

### Enrichment analysis of DEGs

**Figure 3:**
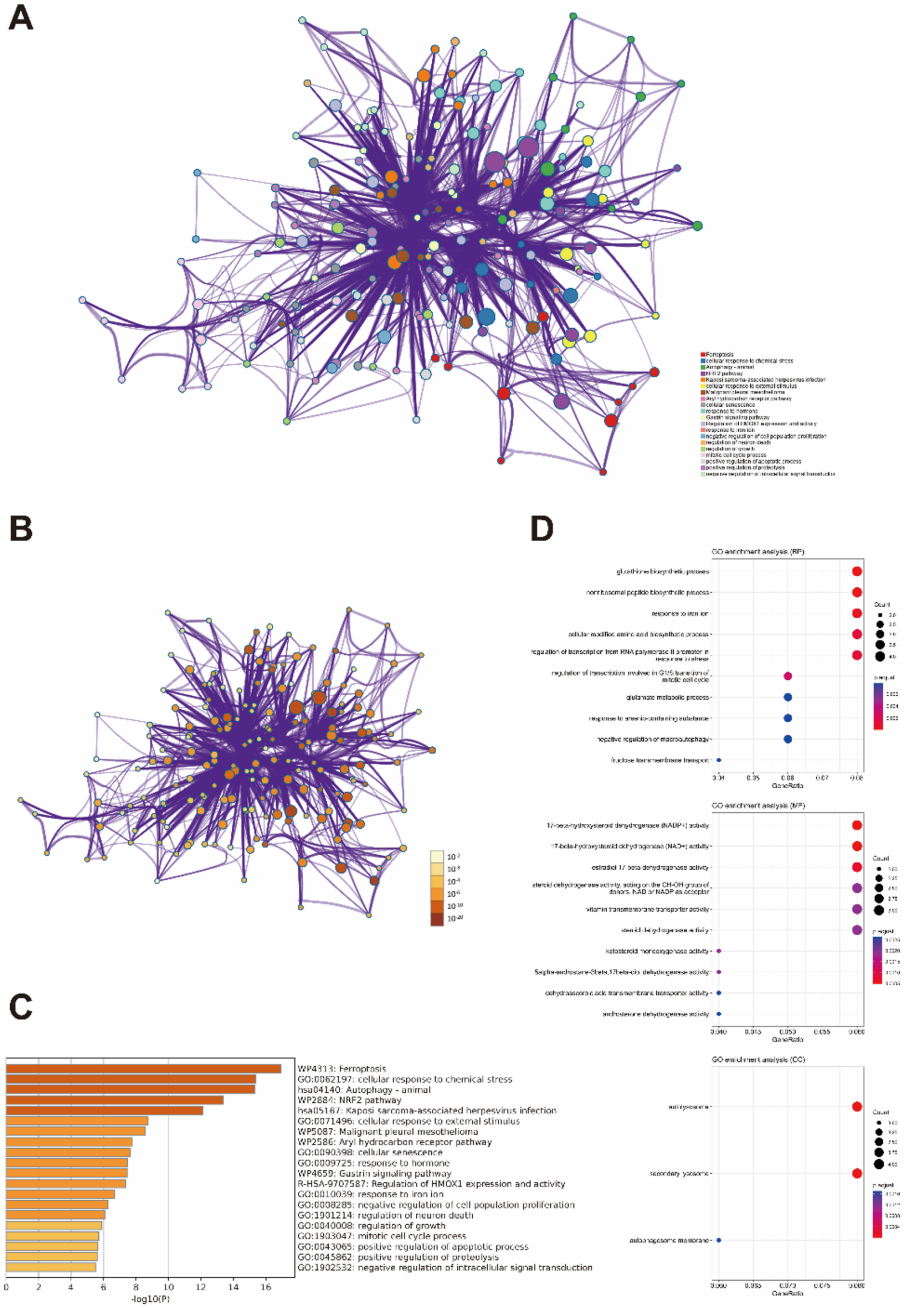
Enrichment analysis of DEGs. (**A**,**B**) Network of enriched terms with Metascape. software. (**C**) The bar chart of the top biological pathways by the Metascape software. (**D**) Go enrichment analysis of biological process, cell components and molecular function.

We investigated the biological mechanisms of 50 ferroptosis-related genes mentioned above using the GO and KEGG pathway enrichment analyses (Fig. 3A,B). Analysis of Metascape demonstrated that these genes was significantly activated in the cellular response to chemical stress, autophagy, NRF2 pathway and Kaposi sarcoma-associated herpesvirus infection (Fig. 3C).It was shown that the biological processes were remarkably enriched in glutathione biosynthetic process, nonribosomal peptide biosynthetic process, response to iron ion, cellular modified amino acid biosynthetic process and regulation of transcription from RNA polymeraseIIpromoter in response to stress, the cell components were enriched in autolysosome and secondary lysosome, the molecular function were enriched in NAD + or NADP + activity and estradiol 17-β-dehydrogenase activity (Fig. 3D).

### PPI network construction and analysis of DEGs

**Figure 4:**
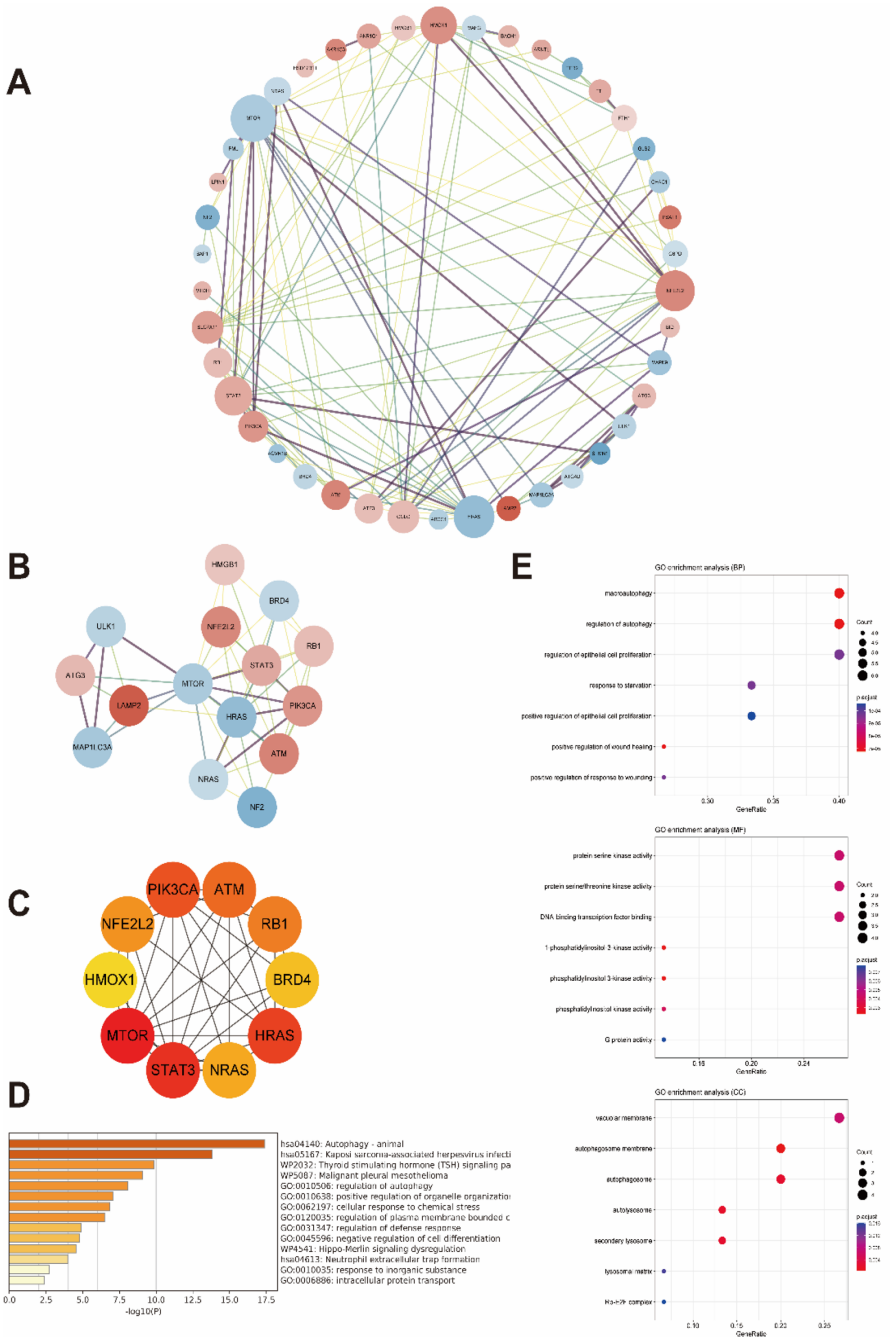
PPI network construction and analysis of DEGs. (**A**) Cytoscape network visualization of the 50 genes according to the STRING online database. (**B**) Cluster of the PPIs identified by the Mcode. (**C**) Select module and the top 10 genes by the CytoHubba. (**D**) The bar chart of the biological pathways in hub modules. (**E**) Go-KEGG enrichment analysis of the hub genes.

To further explore the interactions of DEGs, we imported the 50 genes into the STRING database and constructed a PPI network by the Cytoscape visualization software. We isolated 42 nodes and 122 edges and showed that the larger the degree of nodes, the redder the colour and the larger the points (Fig. 4A). Moreover, we used MCODE to obtain a closest cluster of PPIs (15 nodes, 49 edges, MCODE score ≥ 7) consisting of ATG3, ULK1, MAP1LC3A, LAMP2, MTOR, NRAS, HRAS, STAT3, NFE2L2, HMGB1, BRD4, RB1, PIK3CA, ATM, NF2 (Fig. 4B). Meanwhile, using the CytoHubba to select module and the top 10 genes were taken as the hub genes consisting of PIK3CA, ATM, RB1, BRD4, HRAS, NRAS, STAT3, MTOR, HMOX1, NFE2L2 (Fig. 4C). The PIK3CA, ATM, RB1, BRD4, HRAS, NRAS, STAT3, MTOR, NFE2L2 mined by the two modules were identical. Furthermore, the functional enrichment analysis for the cluster using Metascape and HiPlot showed that the hub genes were mainly involved in autophagy, phosphatidylinositol kinase activity, Kaposi sarcoma-associated herpesvirus infection and Thyroid stimulating hormone (TSH) signaling pathway (Fig. 4D,E).

### Gene-miRNA network construction and analysis

**Figure 5:**
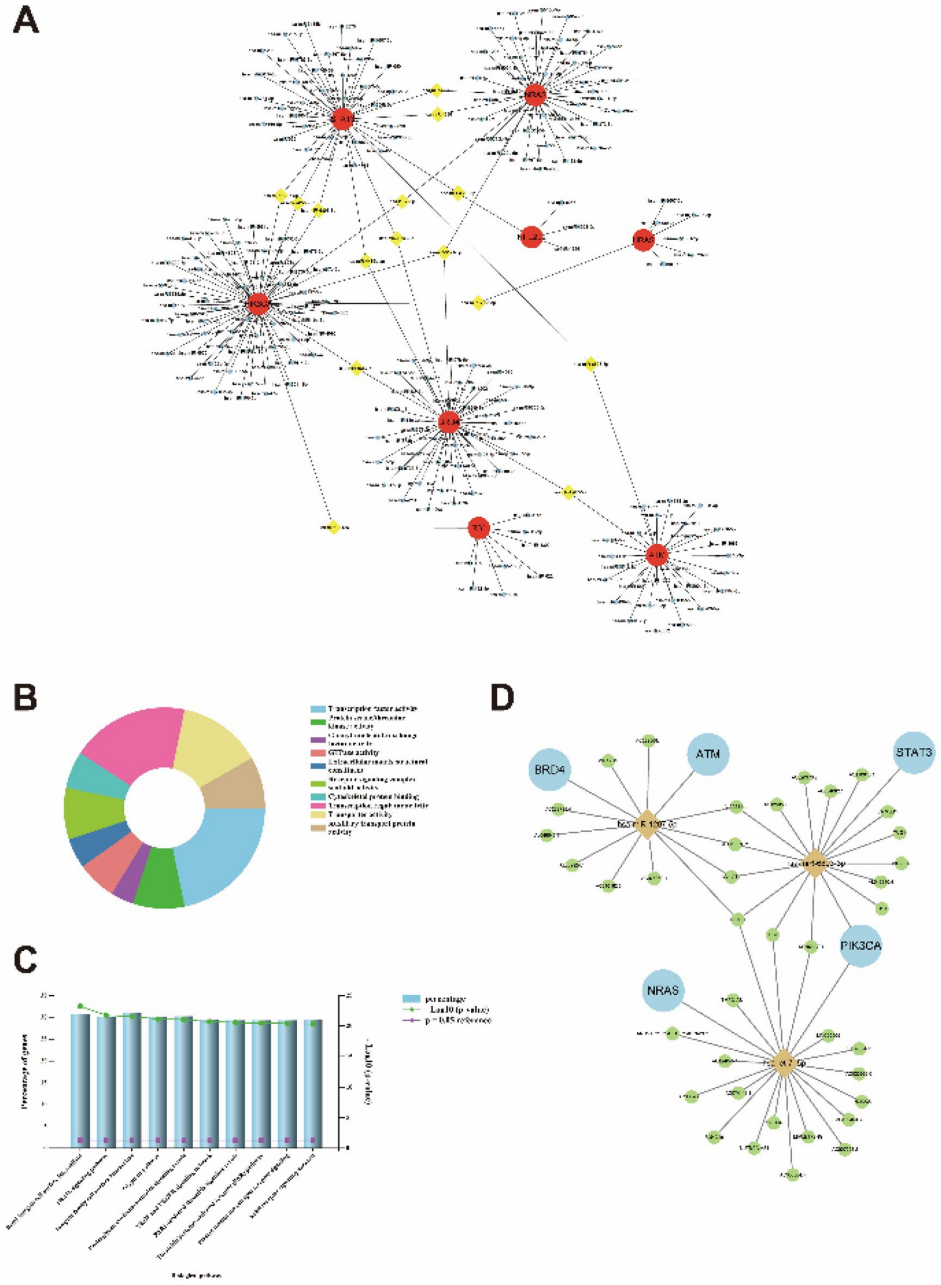
Gene-miRNA and ceRNA network construction. (**A**) Interaction network between hub genes and their targeted miRNAs. Genes were colored in red, miRNAs were colored in blue and the cross-linked genes were colored in yellow. (**B**) Functional enrichment analysis of hub genes related miRNAs by Funrich. (**C**) Biological pathways of the enriched miRNAs. (**D**) The construction of ceRNA network based on the lnRNAs-miRNAs-genes.

We screened 9 hub genes and performed gene-miRNA network by using miRWalk 2.0 software. 8 genes, except MTOR, were modulated and crosslinked by 238 miRNAs (Fig. 5A). The miRNAs with higher amounts of cross-linked target genes (≥ 2) are shown (Table 4). Functional enrichment analysis of 238 miRNAs by Funrich indicated that the molecular function was significantly enriched in transcription factor or regulator activity, protein serine/threonine kinase activity and a series of receptors or transporters activity (Fig. 5B). The biological pathways were enriched in the Integrin family cell surface interaction, TRAIL signaling pathway and proteoglycan syndecan-mediated signaling events (Fig. 5C).

**Table 4:** The miRNAs with higher amounts of cross-linked target genes.

miRNA	Target genes	count
miR-1287-5p	ATM, BRD4	2
miR-6825-5p	ATM, STAT3	2
miR-6735-5p	BRD4, STAT3	2
miR-4436b-3p	BRD4, STAT3	2
miR-3680-3p	STAT3, NFE2L2	2
miR-6857-5p	BRD4, PIK3CA	2
miR-6893-3p	STAT3, PIK3CA	2
miR-6505-5p	STAT3, PIK3CA	2
miR-1227-5p	STAT3, PIK3CA	2
miR-4686	STAT3, NRAS	2
miR-4270	STAT3, NRAS	2
miR-6868-3p	PIK3CA, HRAS	2
let-7i-5p	PIK3CA, NRAS	2
miR-12126	RB1, PIK3CA	2
miR-3191-5p	PIK3CA, NRAS, BRD4	3

### ceRNA network construction and analysis

The corresponding lncRNAs of miRNAs were predicted with StarBase. Even though 15 miRNAs had higher amounts of cross-linked genes (≥ 2), only hsa-miR-1287-5p, hsa-miR-6893-3p and hsa-let-7i-5p had the upstream lncRNAs with higher reliability. The miRNAs cross-linked target lncRNAs and their type are shown (Tables 5, 6, 7). After cross-linking, 5 hub genes were predicted and 38 lncRNAs targeting 3 key miRNAs were revealed, 6 lncRNAs were predicted targeting two or three differential miRNAs included AL022311.1, AC016876.2, MALAT1, NEAT1, XIST and KCNQ1OT1. Based on the above, only NEAT1 served as a key lncRNA that predicted interacting with 3 miRNAs and 5 hub genes, the ceRNA network was constructed (Fig. 5D).

**Table 5:** The hsa-miR-1287-5p cross-linked target lncRNAs and their type.

miRNA	lncRNA	lncRNA type
hsa-miR-1287-5p	AL031282.2	processed_transcript
hsa-miR-1287-5p	AC239798.4	processed_transcript
hsa-miR-1287-5p	AC239868.1	sense_overlapping
hsa-miR-1287-5p	AL359924.1	antisense
hsa-miR-1287-5p	AL049543.1	antisense
hsa-miR-1287-5p	NEAT1	lincRNA
hsa-miR-1287-5p	MALAT1	lincRNA
hsa-miR-1287-5p	AC026362.1	processed_transcript
hsa-miR-1287-5p	MIR17HG	processed_transcript
hsa-miR-1287-5p	AC040162.3	lincRNA
hsa-miR-1287-5p	AC016876.2	processed_transcript
hsa-miR-1287-5p	AL022311.1	sense_overlapping

**Table 6:** The hsa-miR-6893-3p cross-linked target lncRNAs and their type.

miRNA	lncRNA	lncRNA type
hsa-miR-6893-3p	DNM3OS	antisense
hsa-miR-6893-3p	AC084082.1	lincRNA
hsa-miR-6893-3p	H19	processed_transcript
hsa-miR-6893-3p	KCNQ1OT1	antisense
hsa-miR-6893-3p	NEAT1	lincRNA
hsa-miR-6893-3p	MALAT1	lincRNA
hsa-miR-6893-3p	AL049840.4	sense_intronic
hsa-miR-6893-3p	AC116913.1	antisense
hsa-miR-6893-3p	AC135050.6	sense_intronic
hsa-miR-6893-3p	AC020978.7	sense_overlapping
hsa-miR-6893-3p	MIR22HG	lincRNA
hsa-miR-6893-3p	AC016876.2	processed_transcript
hsa-miR-6893-3p	TUG1	bidirectional_promoter_lncRNA
hsa-miR-6893-3p	AL022311.1	sense_overlapping
hsa-miR-6893-3p	XIST	lincRNA

**Table 7:** The hsa-let-7i-5p cross-linked target lncRNAs and their type.

miRNA	lncRNA	lncRNA type
hsa-let-7i-5p	ZNF436-AS1	antisense
hsa-let-7i-5p	AC074117.1	antisense
hsa-let-7i-5p	AC124045.1	lincRNA
hsa-let-7i-5p	SNHG4	lincRNA
hsa-let-7i-5p	LINC00265	lincRNA
hsa-let-7i-5p	AC023632.6	TEC
hsa-let-7i-5p	NUTM2A-AS1	antisense
hsa-let-7i-5p	KCNQ1OT1	antisense
hsa-let-7i-5p	NEAT1	lincRNA
hsa-let-7i-5p	AP000766.1	lincRNA
hsa-let-7i-5p	AC006064.5	sense_intronic
hsa-let-7i-5p	LINC02381	lincRNA
hsa-let-7i-5p	TMPO-AS1	antisense
hsa-let-7i-5p	AC109460.3	processed_transcript
hsa-let-7i-5p	ARHGAP27P1-BPTFP1-KPNA2P3	processed_transcript
hsa-let-7i-5p	SNHG16	processed_transcript
hsa-let-7i-5p	AC007228.2	lincRNA
hsa-let-7i-5p	MIRLET7BHG	lincRNA
hsa-let-7i-5p	XIST	lincRNA

### Validating the expression of related genes in PD models

**Figure 6:**
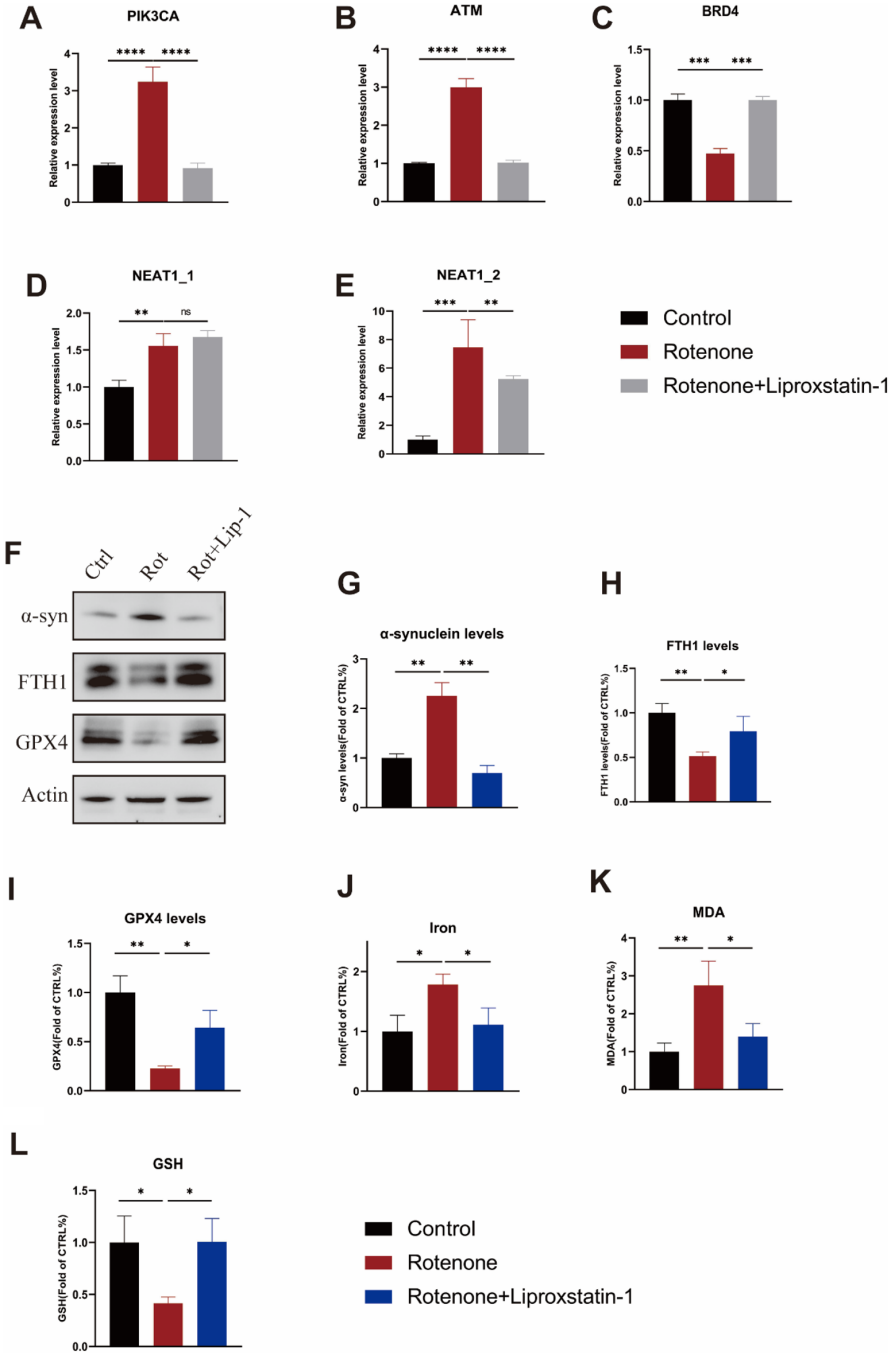
Validation of the DEGs in PD models. (**A**–**E**) The mRNA relative expression levels of PIK3CA, ATM, BRD4, NEAT1 in PD cell culture model in vitro constructed by SH-SY5Y cell using rotenone. (**F**–**I**) Western blot analyses of α-synuclein, FTH1 and GPX4 in SH-SY5Y cells treated Rotenone, Actin was used as an endogenous control. (**J**–**L**) The levels of iron, MDA and GSH in SH-SY5Y cells treated rotenone were determined by using commercial assay kits (*p < 0.05, **p < 0.01, ***p < 0.001, ****p < 0.0001).

The filtered biomarkers, including three hub genes (PIK3CA, BRD4, ATM) and the key lncRNA (NEAT1) with two transcripts, were verified using qRT-PCR. We constructed in vitro PD models based on SH-SY5Y cell line and rotenone, the PD-related neurotoxins. In rotenone groups, the expression levels of PIK3CA and ATM were visibly upregulated and the BRD4 were slightly downregulated to the control. After the ferroptosis special inhibitor liproxstatin-1 added, the higher levels of PIK3CA and ATM with rotenone could be reversed as well as the lower levels of BRD4 (Fig. 6A–C). Interestingly, compared with the hub genes, the expression levels of NEAT1 were significantly upregulated in the neurotoxic groups and could not be rescued by liproxstatin-1 (Fig. 6D). Moreover, the NEAT1_2 levels in rotenone group also showed a higher trend and could be rescued by the liproxstatin-1 (Fig. 6E). We next determined the effects of stimulating the α-syn activation in rotenone models. The results showed that in comparison with control group, the expression of α-syn were increased in rotenone group. Liproxstatin-1 treatment also attenuated rotenone induced the aggregation of α-syn (Fig. 6F,G). To further confirm the ferroptosis in rotenone-treated SHSY5Y cells, GPX4 and FTH1 expressions, iron, MDA and GSH levels, several biomarkers that occur during ferroptosis were examined. As shown in figures (Fig. 6F,H–L), compared with the control group, rotenone exposure increased the content of MDA and iron, the GSH levels or the expressions of GPX4 and FTH1 were significantly consistent decreased. Taken together, these results further confirmed that the NEAT1-PIK3CA/ATM ceRNA network may be activated and directly contacted with the ferroptosis induced by α-syn in PD models.

## Discussion

Ferroptosis as a new cell death pathway has been reported the potential links to loss of Dopaminergic neurons and a number of ferroptosis features are commonly described in living or postmortem PD patients^13^. The aggregated of α-syn in PD pathology has long been investigated, but there is a strong rationale for involvement of the funtion of α-syn in ferroptosis recently. While iron can bind to α-syn oligomers in neurons induce iron-dependent oxidation^14^, the α-syn may also mediates iron metabolism by interacting with the related proteins. The mechanism in which α-syn is proposed to modulate iron import by facilitating the uptake of transferrin1-bound iron and upregulating the iron transport protein DMT1^15,16^. Besides, the aggregated of α-syn may mobilize the cellular iron stores through impairing ferritinophagy^17^. Furthermore, multiple studies report the lipid peroxidation related composition, such as PUFAs,4-HNE and AA, also link with the modification or formation of toxic oligomers^6^, suggesting a possible role of α-syn in lipid metabolism. However, the precise molecular mechanism of α-syn leading to dopaminergic neuronal death through ferroptosis remains unclear.

The present study used microarray data and bioinformatic techniques further explored the mechanisms of α-syn promoting ferroptosis in PD patients. We first obtained the 50 DEGs from the intersection of the datasets GSE49036 and FerrDb, including 27 upregulated genes and 23 downregulated genes. GO and KEGG enrichment analysis, eliminated the interferences, revealed that these DEGs were significantly involved in up or downstream whereby GSH and NRF2 pathway. The phosphatidylinositol kinase activity and the pathways related with lipid metabolism may play a pivotal role^18^. Moreover, the regulatory ceRNA network were successfully constructed, including 5 hub genes,3 miRNAs and 38 lncRNAs. The changes in those genes expression after rotenone and liproxstatin-1 by rescue were identified by qRT-PCR, suggested that the α-syn may influence ferroptosis in the ceRNA network and validated the reliability of the integrated data analysis. We also provided several genes that had not yet been mentioned in the area of PD and ferroptosis, which might be helpful to further illustrate the mechanism of synucleinopathy from the perspective of bioinformatics.

The results showed that the hub genes were significantly enriched in NRF2 signalling pathway which is considered to be an important regulatory factor for ferroptosis. Increased oxidative stress can promote the nuclear translocation of NRF2 and interacts with antioxidant response elements (AREs) resulting in transcriptional activation of ferroptosis moleculars, including NAD(P)H, HO-1, GPX4, SLC7A11/xCT and several iron transporters^19,20^. Our study also provided the evidence that the GSH synthesis and iron or lipid metabolsm related to NRF2 were enriched consistent with the above. Previous study indicated that Nrf2 KO with α-Syn stereotaxic delivery mice showed the dystrophic dendrites and increased neuroinflammation which is largely linked to the PD pathological feature^21^. Meanwhile, the NRF2 loss may enhances the α-Syn phosphorylation, which causes oligomerization and eventual convert the α-Syn into insoluble aggregates^22^. The discovery that there is a vicious cycle of α-syn overexpression, NRF2 suppression, and enhanced ferroptotic neuronal death that could be a key driver of parkinsonian phenotypes^23^. This may explain why the α-syn can accentuate ferroptosis of PD pathology via suppression of NRF2 and if there is a physical interaction between NRF2 and α-syn will be interesting to pursue.

The recently affirmed role of selective autophagy such as ferritinophagy and lipophagy in driving cells toward ferroptotic demonstrated the accuracy of our results. The mechanism of the selective autophagy is the specific autophagy receptors function by interacting simultaneously with pidated LC3 or GABARAP subfamily proteins^24^. Excessive autophagy may impair lysosomal activity and promote ferroptosis, For example, the NCOA4-mediated ferritinophagy can induce the iron accumulation, the RAB7A-mediated lipophagy, SQSTM1-mediated clockophagy and HSP90-mediated CMA (D) may promote the lipid peroxidation in ferroptosis^25^. The interaction of them is also modulated by the upstream regulators of PIK3C3 complex contains the catalytic subunit of phosphatidylinositol 3-kinase^26^. Meanwhile, we found in the present study that the key modules identified by MCODE of PPI were significantly enriched in macroautophagy, autophagosome membrane or the regulation of autophagy and involved in the activity of phosphatidylinositol 3-kinase. As well known, the PI3K/Akt/mTOR signaling pathway was closely related with oxidative stress and the Akt can mediate the PI3K to play a key role in the pathogenesis of PD^27^. Abnormal α-syn aggregation participates in PD is associated with the involvement of TLR4/ PI3K/Akt/NF-κB signaling in neuroinflammation^28^. Therefore, the PI3K/Akt pathway may play an key role in ferroptosis following the α-syn stimulation.

The genes of interest were identified by the key module analyses with the highest MCODE scores and the top10 of CytoHubba, nine overlapped genes, included PIK3CA, ATM, BRD4, RB1, STAT3, NRAS, NFE2L2, HRAS and MTOR were selected as the most significant hub genes. PIK3CA is a major regulators of numerous protein kinases which plays key roles in glutamate oxidative stress-induced cell death in primary cerebrocortical neurons. It has been demonstrated that the protective roles of PIK3CA inhibitors against ferroptosis triggered by glutamate oxytosis and elucidated the potential therapeutic value in PD patients^29^. The DNA damage response serine/threonine kinase ATM was also found to be essential for iron metabolism. ATM induced ferroptosis by decreasing the expression of iron regulators involved in iron storage (ferritin heavy and light chain, FTH1 and FTL) and export (ferroportin, FPN1), moreover, an unexpected ATM-Ferritin/FPN1 regulatory axis as original determinants of ferroptosis through regulating labile iron levels were identified^30^. BRD4 belongs to the bromodomain and extraterminal domain (BET) protein family which can recognize acetylation sites and recruit transcription factors. A previous study suggested that targeting BRD4 may confer a clinical benefit in proliferation of cancer cells via the increase in ferritinophagy and enhanced the ferroptosis^31^, suppression of BRD4 may be associated with the pathogenesis of PD. RB1 and STAT3 are all signal key factors of transcription, in which RB1 is a corepressor of G1/S checkpoint while the STAT3 serves as a activator to enhance the inflammatory response. The negative status of two proteins promotes the occurrence of ferroptosis, especially STAT3, could improve the processes associated with ferroptosis by regulating SLC7A11^32,33^. NRAS and HRAS are the key components of RAS signaling pathways originating from the surface receptors. Emerging studies have provided evidence that the expression of RAS family related genes were significantly more susceptible to loss of neurons viability upon treatment with ferroptosis inducers^34^. Our results showed that the expression of PIK3CA, ATM, RB1, STAT3 and NFE2L2 was upregulated, while BRD4, NRAS, HRAS and MTOR expression was downregulated. The PIK3CA or ATM belongs to the drivers of ferroptosis in the FerrDB database and the BRD4 belongs to the suppressor. These data suggest that the three hub genes may play a significant role in ferroptosis upstream that caused by the α-syn.

The miRNAs are short chain non-coding RNA that can inhibit or degrade the translation of the downstream target genes. In our study, we found eight hub genes could successfully forecast 238 miRNAs and also identified 15 miRNAs targeting at least two genes. Among these miRNAs, only hsa-miR-1287-5p, hsa-miR-6893-3p and hsa-let-7i-5p could predict the relevant lncRNAs. Previous studies on these miRNAs and PD were limited, miR-1287-5p plays critical roles in multiple cancers such as hepatocellular carcinoma, osteosarcoma and breast cancer that involved in pro-inflammatory and antioxidant pathway like GPX4^35^. The miR-6893-3p may regulate IL-6, IGF-1 and VEGF in multiple myeloma or monoclonal gammopathy^36^. Several reults demonstrated the hsa-let-7i-5p could target the fatty acid and lipid metabolism pathway^37^ which consistent with our findings on interaction with PIK3CA. Funrich enrichment analysis indicated that the molecular function was significantly enriched in transcription factor or regulator activity, protein serine/threonine kinase activity and a series of receptors or transporters activity. The biological pathways enriched included the Integrin family cell surface interaction, TRAIL signaling pathway and proteoglycan syndecan-mediated signaling events. Further researches should be carried out to pinpoint the unique molecular mechanism.

lncRNAs refers to a type of long length noncoding RNA that affect the downstream miRNAs but not translate into proteins. In this study, five hub genes (PIK3CA, ATM, BRD4, STAT3, NRAS) and three miRNAs (hsa-miR-1287-5p, hsa-miR-6893-3p, hsa-let-7i-5p) were successfully constructed in the ceRNA network. They were highly correlated with 38 lncRNA and six of them targeting at least two miRNAs, included AL022311.1, AC016876.2, MALAT1, NEAT1, XIST and KCNQ1OT1. The nuclear paraspeckle assembly transcript 1 (NEAT1) has been showed to be involved in the regulation of cell cycle, proliferation and migration of tumor, apoptosis and ferroptosis. NEAT1 has two transcripts contains NEAT1_1 (3.7 kb) and NEAT1_2 (23 kb) which act as sponges to absorb miRNA thereby promoting the target genes express^38^. A previous research found that NEAT1 could competitively bind miR362-3p and thus leads to inhibition of Myo-inositol oxygenase (MIOX), the axis was regulated by the p53 and thereby increasing the sensitivity to ferroptosis^39^. Our findings identified that only NEAT1 predicted three target miRNAs and served as a maincenter linking five key genes together. It may be potentially used as a novel biomarker of α-syn promoting ferroptosis in PD patients.

In order to further affirm our results, we performed experiments in vitro by generating the PD models with neurotoxins. We used SH-SY5Y cells and developed by introducing rotenone to validate the integrated data. The qRT-PCR analysis in our own samples showed that PIK3CA and ATM were significantly upregulated in rotenone group, whereas BRD4 were downregulated. Research studies have manifested that in the rotenone model, α-Syn were observed in the surviving dopaminergic neuron, however, the MPP + and other neurotoxin models lack the formation of Lewy Bodies^40^. Our western blot analysis consistent showed that the rotenone could stimulate the aggregation of α-Syn thereby causing the ferroptosis. Furthermore, liproxstatin-1, a specific ferroptosis inhibitor, can ameliorate the cells injury and reduce the expression of PIK3CA and ATM. It may be that ferroptosis inhibitor could suppress the toxic α-syn oligomers via the mechanism of aggregate-membrane interaction^8^. Therefore, PIK3CA and ATM may serve as new hub genes for α-Syn promoting ferroptosis. We also found the key lncRNAs NEAT1, whether NEAT1_1 or NEAT1_2, was upregulated in the rotenone groups. The expression of NEAT1, especially NEAT1_2, in rotenone group were significant higher compared with the control. Together, we proposed α-Syn as a key contributor to ferroptosis progression which may involve in NEAT1-PIK3CA/ATM ceRNA network. These demostrated the hub genes and the ceRNA may act independently on ferroptosis but not other cell death mechanism and broaden new insights that targeting the ceRNA network can be developing strategies for PD therapy (Supplementary Figure).

Despite the significant results obtained in our study, there were several limitations. First, our data are not from high-throughput sequencing but from single microarray databases, the number of samples was not large enough which may contribute to a high false-positive rate and one-sided results. Moreover, although we analyzed the expression of related genes and lncRNAs in PD cell models, the relationship between them and other unknown molecules are still undetected, single-cell RNA-Seq or other methods are necessary for verifying our results and illustrating the roles of PIK3CA, ATM and NEAT1 in PD. In addition, functional experiments based on transgenic PD models in vivo and induced pluripotent stem cells from PD patients are need to further verify the expression of current genes protein level in related mechanisms.

In summary, our current study provided a reliable comprehensive analysis of combining the α-syn and ferroptosis based on the gene expression profiling. The identified DEGs revealed core networks and molecules significantly associated with the α-syn aggregation driving ferroptosis in SN of PD patients. The above view were all strongly correlated with NRF2, PI3K and autophagy signaling pathway. Our results will help enhance the cognition of the internal connection between the two independent death patterns, ferroptosis and autophagy. We believe that the genes verified by our findings may become specific diagnostic markers as well as provide promising targets for genetic therapy for PD patients in the future.

### Supplementary Information

Supplementary Information.

## Data Availability

The data can be found in FerrDb database (http://www.zhounan.org/ferrdb/) and the GEO database (https://www.ncbi.nlm.nih.gov/geo/query/acc.cgi? acc = GSE49036). Further inquiries can be directed to the corresponding author.

## References

[1] Fearnley JM, Lees AJ (1991). Ageing and Parkinson's disease: Substantia nigra regional selectivity. Brain..

[2] Pankratz N (2009). Genomewide association study for susceptibility genes contributing to familial Parkinson disease. Hum. Genet..

[3] Masuda-Suzukake M (2013). Prion-like spreading of pathological α-synuclein in brain. Brain.

[4] Dixon SJ (2012). Ferroptosis: An iron-dependent form of nonapoptotic cell death. Cell.

[5] Stockwell BR (2017). Ferroptosis: A regulated cell death nexus linking metabolism, redox biology, and disease. Cell.

[6] Mahoney-Sánchez L, Bouchaoui H, Ayton S, Devos D, Duce JA, Devedjian JC (2021). Ferroptosis and its potential role in the physiopathology of Parkinson's Disease. Prog. Neurobiol..

[7] Guiney SJ, Adlard PA, Bush AI, Finkelstein DI, Ayton S (2017). Ferroptosis and cell death mechanisms in Parkinson's disease. Neurochem. Int..

[8] Angelova PR (2020). Alpha synuclein aggregation drives ferroptosis: An interplay of iron, calcium and lipid peroxidation. Cell Death Differ..

[9] Dijkstra AA (2015). Evidence for immune response, axonal dysfunction and reduced endocytosis in the substantia Nigra in early stage Parkinson's disease. PLoS One.

[10] Kanehisa M, Goto S (2000). KEGG: Kyoto encyclopedia of genes and genomes. Nucleic Acids Res..

[11] Kanehisa M (2019). Toward understanding the origin and evolution of cellular organisms. Protein Sci..

[12] Kanehisa M, Furumichi M, Sato Y, Kawashima M, Ishiguro-Watanabe M (2023). KEGG for taxonomy-based analysis of pathways and genomes. Nucleic Acids Res..

[13] Martin-Bastida A (2017). Brain iron chelation by deferiprone in a phase 2 randomised double-blinded placebo controlled clinical trial in Parkinson's disease. Sci. Rep..

[14] Deas E (2016). Alpha-synuclein oligomers interact with metal ions to induce oxidative stress and neuronal death in Parkinson's disease. Antioxid. Redox Signal..

[15] Baksi S, Tripathi AK, Singh N (2016). Alpha-synuclein modulates retinal iron homeostasis by facilitating the uptake of transferrin-bound iron: Implications for visual manifestations of Parkinson's disease. Free Radic. Biol. Med..

[16] Bi M, Du X, Jiao Q, Liu Z, Jiang H (2020). α-Synuclein regulates iron homeostasis via preventing Parkin-mediated DMT1 ubiquitylation in Parkinson's disease models. ACS Chem. Neurosci..

[17] Baksi S, Singh N (2017). α-Synuclein impairs ferritinophagy in the retinal pigment epithelium: Implications for retinal iron dyshomeostasis in Parkinson's disease. Sci. Rep..

[18] Sun WY (2021). Phospholipase iPLA_2_β averts ferroptosis by eliminating a redox lipid death signal. Nat. Chem. Biol..

[19] Yamamoto M, Kensler TW, Motohashi H (2018). The KEAP1-NRF2 system: A thiol-based sensor-effector apparatus for maintaining redox homeostasis. Physiol. Rev..

[20] Abdalkader M, Lampinen R, Kanninen KM, Malm TM, Liddell JR (2018). Targeting Nrf2 to suppress ferroptosis and mitochondrial dysfunction in neurodegeneration. Front. Neurosci..

[21] Lastres-Becker I, Ulusoy A, Innamorato NG, Sahin G, Rábano A, Kirik D, Cuadrado A (2012). α-Synuclein expression and Nrf2 deficiency cooperate to aggravate protein aggregation, neuronal death and inflammation in early-stage Parkinson's disease. Hum. Mol. Genet..

[22] Anandhan A (2021). NRF2 loss accentuates Parkinsonian pathology and behavioral dysfunction in human α-synuclein overexpressing mice. Aging Dis..

[23] Anandhan A, Chen W, Nguyen N, Madhavan L, Dodson M, Zhang DD (2022). α-Syn overexpression, NRF2 suppression, and enhanced ferroptosis create a vicious cycle of neuronal loss in Parkinson's disease. Free Radic. Biol. Med..

[24] Johansen T, Lamark T (2020). Selective autophagy: ATG8 family proteins, LIR motifs and cargo receptors. J. Mol. Biol..

[25] Liu J, Kuang F, Kroemer G, Klionsky DJ, Kang R, Tang D (2020). Autophagy-dependent ferroptosis: Machinery and regulation. Cell Chem. Biol..

[26] Turco E, Fracchiolla D, Martens S (2020). Recruitment and activation of the ULK1/Atg1 kinase complex in selective autophagy. J. Mol. Biol..

[27] Rai SN (2019). The role of PI3K/Akt and ERK in neurodegenerative disorders. Neurotox. Res..

[28] Zhong Z (2021). Fecal microbiota transplantation exerts a protective role in MPTP-induced Parkinson's disease via the TLR4/PI3K/AKT/NF-κB pathway stimulated by α-synuclein. Neurochem. Res..

[29] Kang Y, Tiziani S, Park G, Kaul M, Paternostro G (2014). Cellular protection using Flt3 and PI3Kα inhibitors demonstrates multiple mechanisms of oxidative glutamate toxicity. Nat. Commun..

[30] Chen PH (2020). Kinome screen of ferroptosis reveals a novel role of ATM in regulating iron metabolism. Cell Death Differ..

[31] Sui S, Zhang J, Xu S, Wang Q, Wang P, Pang D (2019). Ferritinophagy is required for the induction of ferroptosis by the bromodomain protein BRD4 inhibitor (+)-JQ1 in cancer cells. Cell Death Dis..

[32] Louandre C (2015). The retinoblastoma (Rb) protein regulates ferroptosis induced by sorafenib in human hepatocellular carcinoma cells. Cancer Lett..

[33] Qiang Z, Dong H, Xia Y, Chai D, Hu R, Jiang H (2020). Nrf2 and STAT3 alleviates ferroptosis-mediated IIR-ALI by regulating SLC7A11. Oxid. Med. Cell Longev..

[34] Schott C, Graab U, Cuvelier N, Hahn H, Fulda S (2015). Oncogenic RAS mutants confer resistance of RMS13 rhabdomyosarcoma cells to oxidative stress-induced ferroptotic cell death. Front. Oncol..

[35] Xu Z, Chen L, Wang C, Zhang L, Xu W (2021). MicroRNA-1287-5p promotes ferroptosis of osteosarcoma cells through inhibiting GPX4. Free Radic. Res..

[36] Li J, Zhang M, Wang C (2020). Circulating miRNAs as diagnostic biomarkers for multiple myeloma and monoclonal gammopathy of undetermined significance. J. Clin. Lab. Anal..

[37] Langi G, Szczerbinski L, Kretowski A (2019). Meta-analysis of differential miRNA expression after bariatric surgery. J. Clin. Med..

[38] Naganuma T, Nakagawa S, Tanigawa A, Sasaki YF, Goshima N, Hirose T (2012). Alternative 3'-end processing of long noncoding RNA initiates construction of nuclear paraspeckles. EMBO J..

[39] Zhang Y, Luo M, Cui X, O'Connell D, Yang Y (2022). Long noncoding RNA NEAT1 promotes ferroptosis by modulating the miR-362-3p/MIOX axis as a ceRNA. Cell Death Differ..

[40] Schober A (2004). Classic toxin-induced animal models of Parkinson's disease: 6-OHDA and MPTP. Cell Tissue Res..

