# Supplementary material for: Identifying the potential genes in alpha synuclein driving ferroptosis of Parkinson’s disease
Source: Sci Rep. 2023 Oct 6;13:16893. doi: 10.1038/s41598-023-44124-4 (PMC10558439; doi:10.1038/s41598-023-44124-4)
Supplement: Supplementary file 1 — Supplementary Information. [file 41598_2023_44124_MOESM1_ESM.pdf]

Figure 6 F

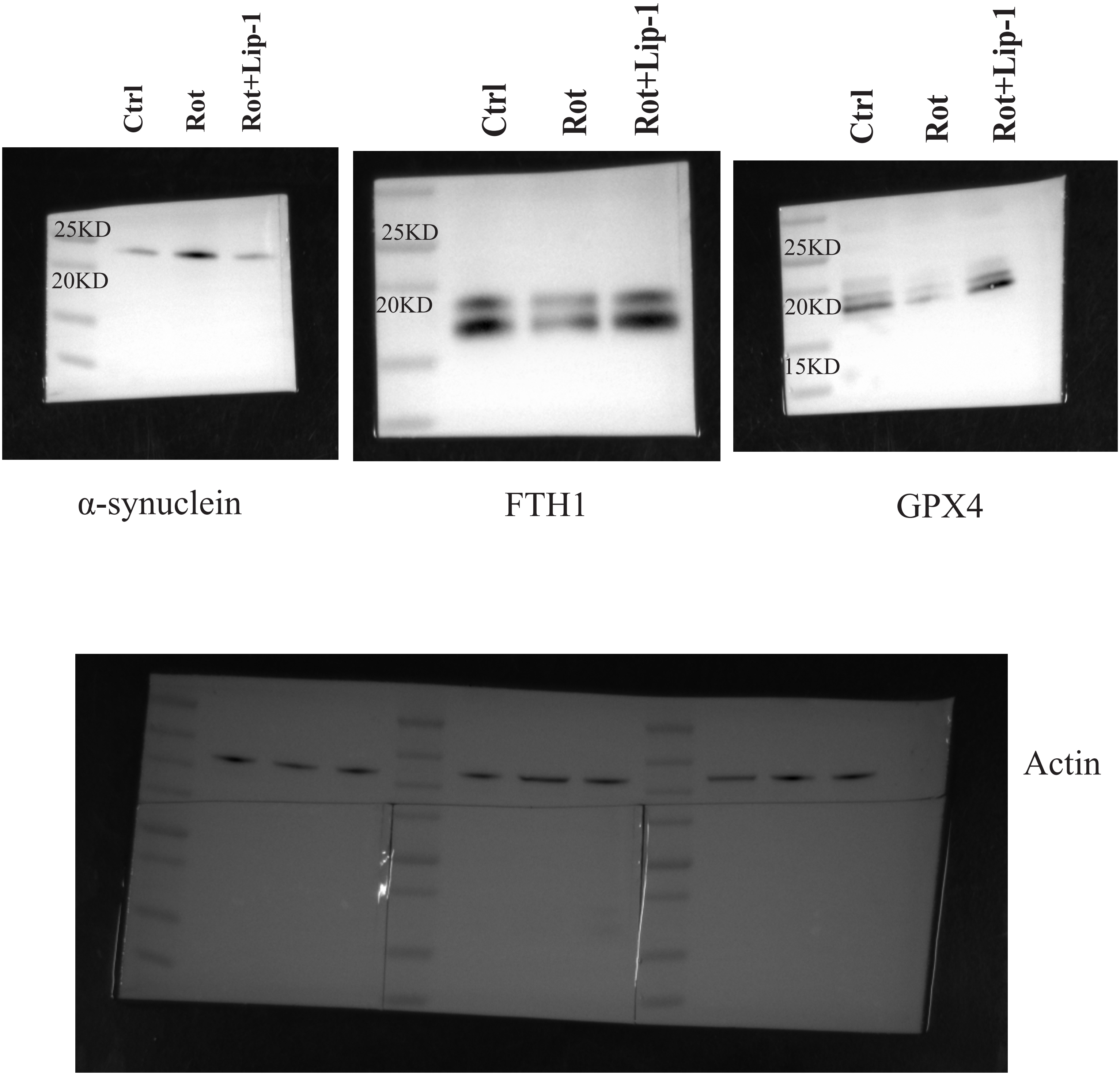

Put them together and take pictures:
Each group were from three different samples, The first three gels were Ctrl, the three gels in the middle were Rot group and the last three gels were Rot+Lip-1
